# Restaurant Owners’ Perspectives on a Voluntary Program to Recognize Restaurants for Offering Reduced-Size Portions, Los Angeles County, 2012

**DOI:** 10.5888/pcd11.130310

**Published:** 2014-03-20

**Authors:** Lauren Gase, Lauren Dunning, Tony Kuo, Paul Simon, Jonathan E. Fielding

**Affiliations:** Author Affiliations: Lauren Dunning, Tony Kuo, Paul Simon, Jonathan E. Fielding, Los Angeles County Department of Public Health, Los Angeles, California.

## Abstract

**Introduction:**

Reducing the portion size of food and beverages served at restaurants has emerged as a strategy for addressing the obesity epidemic; however, barriers and facilitators to achieving this goal are not well characterized.

**Methods:**

In fall 2012, the Los Angeles County Department of Public Health conducted semistructured interviews with restaurant owners to better understand contextual factors that may impede or facilitate participation in a voluntary program to recognize restaurants for offering reduced-size portions.

**Results:**

Interviews were completed with 18 restaurant owners (representing nearly 350 restaurants). Analyses of qualitative data revealed 6 themes related to portion size: 1) perceived customer demand is central to menu planning; 2) multiple portion sizes are already being offered for at least some food items; 3) numerous logistical barriers exist for offering reduced-size portions; 4) restaurant owners have concerns about potential revenue losses from offering reduced-size portions; 5) healthful eating is the responsibility of the customer; and 6) a few owners want to be socially responsible industry leaders.

**Conclusion:**

A program to recognize restaurants for offering reduced-size portions may be a feasible approach in Los Angeles County. These findings may have applications for jurisdictions interested in engaging restaurants as partners in reducing the obesity epidemic.

## Introduction

Child and adult obesity rates in the United States have increased dramatically in the past 20 years (1–3). Rising obesity rates have paralleled the increase in the proportion of foods consumed outside the home (4). Compared with foods prepared at home, foods consumed away from the home are typically higher in calories, sodium, and fat (4–6). Portion sizes of meals at restaurants have also increased over the past 2 decades (7), and larger portion sizes are linked to people consuming more food and underestimating calorie intake (8,9).

As a strategy for addressing the obesity epidemic, numerous US jurisdictions and organizations have created voluntary public recognition programs for restaurants. Such programs typically offer restaurants publicity, technical assistance, or other incentives if they comply with program standards, which frequently focus on setting limits on nutrient content (eg, calories, fat, sodium) for a small number of meals or items (10,11) or increasing the availability of more healthful options (eg, whole grains, plant-based options) (12,13). Although emerging evidence suggests that voluntary recognition programs are feasible (12,14) and may be acceptable to customers (15,16), few studies have examined the factors that impede or facilitate program participation.

The Los Angeles County (LAC) Department of Public Health (DPH) launched a formative research investigation in January 2012 to study the feasibility of establishing a countywide, voluntary public recognition program for restaurants. DPH sought to support broad participation from restaurants in the region by creating program standards that eliminated the need for nutrient analysis, a documented barrier to program participation (17). Guided by reviews of the literature and information from other jurisdictions, DPH drafted program criteria that focused on offering restaurant customers the option to choose reduced-size portions from the menu (in addition to standard-size menu items) and improving the healthfulness of children’s meals. We sought feedback from restaurant owners on this approach, focusing primarily on understanding the barriers and facilitators to restaurants offering reduced-size portions.

## Methods

### Sample and interview procedures

Because little is known about restaurant owners’ interest in offering and ability to offer reduced-size portions, DPH selected a qualitative approach to study this issue. DPH conducted semistructured qualitative interviews with restaurant owners, operators, presidents, or directors who owned or operated restaurants in LAC (hereafter “restaurant owners”) to gather in-depth information on restaurants’ capacity to offer reduced-size portions. Purposeful recruitment, based on maximum variation sampling principles (18), was applied by defining target restaurant categories by cuisine type, geographic location, and average cost per meal. Independent restaurant owners, or owners of small chains or affiliated groups, were given priority for inclusion (over large chains) on the basis of their potential to provide complete information about the process for making menu changes in their organization.

Recruitment occurred in 2 stages. First, in July 2012, representatives from DPH gave a brief presentation on the proposed restaurant recognition program at a regularly scheduled meeting of the Los Angeles chapter of the California Restaurant Association. At the meeting, DPH recruited volunteers to participate in the interviews. All 7 attendees who were restaurant owners expressed interest in participating in the interview and provided their contact information. Second, desired target restaurant categories were shared with the DPH’s restaurant hygiene inspection program which, through a gradual recruitment process, identified additional participants to respond for restaurant categories we had not yet filled (eg, restaurants located in South Los Angeles, restaurants that serve Asian cuisine). All restaurants were recruited through e-mail or telephone. An interview was scheduled with each owner who agreed to participate.

### Data collection

Semistructured interviews were conducted via telephone by DPH’s interview team during September through November 2012. Each interview used a 34-question scripted interview guide that contained both closed- and open-ended questions organized in 5 sections: restaurant background (eg, size, restaurant type, meal price range), restaurant customers (eg, type and number of customers), current healthful offerings and activities (eg, menu labeling or signage, menu items available in reduced-size portions), ability to meet proposed DPH program standards (eg, feasibility of offering reduced-size portions and more healthful children’s meals), and potential facilitators to program participation (eg, interest in public recognition and technical assistance). When a restaurant owner represented multiple different restaurants, he or she was asked to select 1 restaurant (based on his or her preference) to base answers on. Before field implementation, all study protocols and materials were reviewed and approved by the Los Angeles County Department of Public Health Institutional Review Board.

Each interview lasted approximately 30 to 60 minutes. The interviews were not audiotaped because many prospective interviewees had indicated they would not participate if taping was involved. Each interview was attended by 2 members from the interview team (in addition to the primary interviewer), who took detailed notes to capture responses. At the completion of each interview, the primary interviewer and the 2 members summarized personal impressions and reactions as well as emerging themes about the telephone encounter; these documentations were completed independently and analyzed along with the interview notes.

### Data analysis

Interview data were analyzed by using a 3-stage process. First, the interviewer and 1 of the team members who attended the interview conducted an independent analysis of the interview notes and their personal and theoretical summaries to identify themes that reflected barriers or facilitators to program participation. Each team member then reviewed all of the documentation a second time to assign codes to specific excerpts of text using the constant comparison method (18). Second, team members compared the independently coded themes to develop a consolidated list of themes. The third member of the interview team was consulted if the first 2 could not reach consensus regarding the code. Finally, themes were vetted and finalized with a panel of DPH leadership, restaurant hygiene inspectors, and dietitians.

## Results

Of the 38 restaurant owners initially contacted, 18 (47%) completed an interview. About half (56%) of the respondents were owners or operators of small chains or affiliated groups of restaurants (Table). Together (not including a large national chain, which represented 281 restaurants), the owners we interviewed represented 66 restaurants, each of which served on average 250 customers per day. Data analysis yielded 6 themes related to barriers or facilitators to offering reduced-size portions. Because most owners supported (and reported few barriers to) improving the healthfulness of children’s meals, themes related to this aspect of the proposed program are not reported here.

**Table:** Characteristics of Independent Restaurants and Restaurant Groups as Reported by Restaurant Owners (n = 18), Los Angeles County, 2012

Restaurant Characteristics	No. of Restaurants^a^
**Chain or affiliated group**	
Large chain or affiliated group (>20 individual locations)	1
Small chain or affiliated group (2–19 individual locations)	10
Only 1 location	7
**Service type**	
Quick service	4
Fast casual	4
Sit down	10
**Average price range for a meal, $^b^**	
<10	5
10–15	10
≥15	1
**Type of cuisine^c^**	
American	8
Italian/Mediterranean	2
California cuisine	1
Mexican	5
Asian	2
Vegetarian	2
Soul food	1

a When a restaurant owner represented multiple different restaurants, he or she was asked to select 1 restaurant (based on his or her preference) to base answers on. Although chain restaurants tend to be similar, restaurants within an affiliated group may have varied characteristics.

b Percentages may not add to 100% because only 16 restaurants provided price information.

c Percentages may not add to 100% because 2 restaurants self-identified as more than 1 cuisine type.

### Perceived customer demand is central to menu planning

During the interviews, 17 restaurant owners mentioned the importance of customer demand in menu planning. As stated by 1 owner, “The restaurant owners will do whatever it takes to accommodate the customer.” The perceived type of demand differed depending on the type of customers the restaurant typically served. Half of the restaurant owners interviewed, whose restaurants tended to be more chef-driven or offered specialty healthy cuisine, mentioned that their customers were more health-conscious (eg, interested in ordering lower-calorie items, nutrition information, vegetarian and gluten-free options). Many of the restaurants already offered what they perceived as healthier options or in-house promotions of more healthful foods (eg, healthier product substitutions, messages on the menu about sauces or dressing on the side). Owners of these restaurants generally expressed interest in participating in the proposed voluntary program because they thought their customers would respond favorably to reduced-size portions.

Ten restaurant owners mentioned they did not perceive a strong demand from their customers for healthy food or reduced-size portions. These owners tended to represent restaurants that were quick service, offered meals at lower cost, or served customers who were tourists. They stated that their customers “value large portions,” “don’t want to feel cheated,” and “expected large sizes for certain items.” One restaurant owner commented that customers came to his restaurant “to treat themselves.” Another stated that his customers would generally consider any item labeled as “healthy” as not flavorful. These owners tended to be most concerned about the program. However, most did not rule out participating. Instead, they emphasized the need to wait and see how their customers would react to reduced-size portions and advocated for more public education. One owner stated, “[Customers] are used to having dishes look a certain way. There would have to be educational efforts for our customers.”

The 2 subthemes mentioned above (perceived customer demand versus no customer demand) were not mutually exclusive. For example, some restaurant owners who were more cautious about offering reduced-size portions commented on society’s recent “culture shift” toward healthier options. As stated by 1 owner “[The restaurant] is a place where people go to indulge. [Smaller portions] haven’t been a part of the culture, but with the advent of emphasis on healthy cuisine, [we] are opening ourselves up to it more.”

### Many restaurants already offer multiple portion sizes

Twelve restaurant owners reported that their establishment(s) already offered at least some menu items in multiple portion sizes. The most commonly cited items that were available in multiple sizes were soups, salad, sandwiches, pizzas, and side dishes; only 8 reported offering multiple sizes for entrées. The 3 most commonly mentioned reasons for offering multiple portion sizes were accepted industry practice (eg, a bowl and a cup of soup, a “senior” menu with smaller portions), efforts to facilitate “customer choice” (ie, so customers could mix and match different food offerings), and ability to have more competitive (lower) prices for reduced-size items. No restaurant owners mentioned offering reduced-size portions for health reasons.

### Perceived logistical barriers to offering reduced-size portions

When asked about the possibility of offering reduced-size portions for additional menu items, 12 restaurant owners named logistical challenges. In general, they were particularly concerned about serving reduced-size entrée items, asking questions like “How do you cut a hot dog in half?” or citing presentation barriers (eg, the need to make the food look like it is filling the whole plate, the need for smaller serving vessels). As stated by 1 owner, “Entrées are much harder to cut down as it relates to presentation and perceived customer value.”

Some restaurant owners were resistant to changing their menus or making their menus more complicated (ie, confusing to customers). Some owners also raised preparation challenges, including the need for additional time, staff training, and management oversight to prepare and serve a larger variety of items. Some owners cited lack of knowledge about how to increase healthfulness of meals or reduce portion sizes or both (eg, how reduced-size entrées could be made appealing) as barriers. Many raised concerns about the availability and costs of smaller product sizes from vendors (eg, smaller tortillas, smaller hamburger buns); this was especially true for restaurants that used mostly prepared items. Finally, many restaurant owners noted the potential increase in costs associated with nutrient analysis and calorie postings although such analyses would not be a part of the proposed program. Many owners cited these costs as particularly challenging for small restaurant businesses.

### Concerns about declining revenues when offering reduced-size portions

Six restaurant owners were concerned about the potential negative economic impact of offering reduced-size portions. All owners agreed that reduced-size portions would need to be offered at a lower price (compared with standard sizes) and most believed that many customers would be motivated to choose the smaller portions to spend less money. Many noted that restaurants have fixed costs (eg, staff, space) not associated with portion sizes, so if they sell the same amount of reduced-size items, this would affect their bottom line. As stated by 1 owner, “Smaller portions increase operating costs and the profit margin is too small to offer smaller portions.” To participate, owners would need a way to offset the potential decrease in revenues (eg, by serving more customers, by charging higher prices for other menu items). One restaurant owner proposed charging higher prices on beverages as a way to make up for possible revenue losses due to selling reduced-size portions.

### Healthy eating is the responsibility of the customer

Five restaurant owners emphasized that people have the ultimate responsibility to make healthful food choices. Many owners felt that restaurants have been unfairly targeted as a cause of the obesity epidemic. Owners emphasized the need for further public education and cited the lack of customer demand to facilitate making substantive changes to their menus. As stated by 1 owner, “We need to educate the public about not overeating. We don’t want to babysit everyone. . . . I can pay attention to what I serve, but I have no control over what people are buying.” Similarly, many restaurant owners were resistant to government involvement (ie, regulation) in determining what size portions they should serve.

### Desire to be a socially responsible industry leader

Three restaurant owners mentioned the desire to offer smaller portions for social or personal reasons, including the need to reduce plate waste, increase the health of their community, or because they have personally experienced the negative health effects of obesity or related disease (eg, diabetes). As described by 1 owner, “We also have to set an example. People look to [us to] be a leader because we’ve always been on the forefront of these types of issues.”

## Discussion

Offering reduced-size portions appears to be a feasible approach among restaurants that participated in this study. Restaurant owners reported many potential barriers to offering reduced-size portions, including lack of perceived customer demand, operational barriers related to preparation and serving, unfavorable economics, and the need for customer responsibility in making food choices. However, despite the perceived barriers noted, some owners see demand from customers for more healthful menu items, many currently offer at least some menu items in reduced-size portions, and most indicated interest in learning more about or participating in the proposed restaurant recognition program.

Similar to findings in prior research, our findings of the need to successfully meet perceived customer demand were cited by many restaurant owners as a central driver to menu planning (19,20). Previous studies suggest that customers have come to expect large portion sizes (8) and that restaurants may be reluctant to reduce current portion size because of customer demand for economic value (8,17). Our study, along with previous research, suggests that a successful restaurant recognition program should engage both restaurant owners and customers. If restaurant owners see demand for smaller portions from customers, they may be more willing to offer reduced-size portions. Public health efforts to help demonstrate this demand could include a health marketing or public education campaign that encourages customers to choose smaller portions or increases public awareness of restaurants that provide reduced-size portions. Likewise, there may be opportunities to educate restaurant owners about existing evidence demonstrating public demand for more healthful options and opportunities for sales growth in this arena (21,22). Helping restaurants to see the societal value in providing reduced-size options might further fuel the demand for smaller portion sizes. Finally, further consideration should be given on ways to label reduced-size portions (eg, “healthier-size” or “normal-size”) because prior research suggests that size labeling may influence customer receptivity (23).

Our results suggest the need for targeted engagement and technical assistance with restaurant owners. A successful restaurant recognition program will need to address challenges with food preparation and presentation associated with portion size, found both in this study and previous work (17). Providing one-on-one technical assistance may be a way for public health to address restaurant-specific concerns and facilitate program participation. Similarly, if concerns about loss of profits are substantiated, changes in pricing structure or mechanisms to offset losses may need to be implemented by restaurants. Peer-to-peer recruitment and mentorship, in which restaurant owners are able to exchange success stories, pricing strategies, and troubleshooting tips, may help facilitate program participation and sustainability.

Although this formative work provides critical insights on restaurant owners’ perspectives, its methods are subject to limitations. LAC has a very large number of restaurants, distributed across a wide geographic area. Despite efforts to obtain a diversity of opinions, limited time and staffing constraints precluded comprehensive sampling of all combinations of restaurant categories. Furthermore, sampling methods were designed to gather information from chef-driven independent or small chain restaurants, which are not necessarily representative of all LAC restaurant owners. The opinions expressed by the owners in this study may be more favorable toward the program than the opinions of the general LAC restaurant owner population. Special consideration will need to be given to the potential barriers and facilitators for large chain restaurants, a substantial number of which are represented in the region. Factors that shaped our decision not to focus on them in this study, such as the lack of participation by franchisees in menu planning, indicate the need for further study of large chain–specific barriers and facilitators to participation in voluntary restaurant recognition programs.

Much remains unknown about best practices for administering a successful voluntary recognition program focused on reduced-size portions. Although this qualitative study suggests that this approach for operating a voluntary program may be feasible, further assessment (eg, quantitative survey with a larger sample) of restaurant owners’ and customers’ knowledge, attitudes, and beliefs for program implementation may be warranted.

The study findings serve as a useful starting point for engaging restaurant owners and suggest that a program to recognize restaurants for providing reduced-size portions may be a feasible approach. The lessons learned from this formative work may have applications in other jurisdictions, especially for those interested in establishing similar programs to address nutrition-related issues and obesity in their communities.
